# Prevalence and Correlates for Psychosocial Distress Among In-School Adolescents in Zambia

**DOI:** 10.3389/fpubh.2015.00180

**Published:** 2015-07-16

**Authors:** Seter Siziya, Mazyanga Lucy Mazaba

**Affiliations:** ^1^Department of Clinical Sciences, School of Medicine, Copperbelt University, Ndola, Zambia; ^2^Department of Public Health, School of Health Sciences, University of Lusaka, Lusaka, Zambia; ^3^Immune and Vaccine Preventable Diseases, World Health Organization, Lusaka, Zambia; ^4^Virology Unit, University Teaching Hospital, Ministry of Health, Lusaka, Zambia

**Keywords:** psychosocial distress, social support, physical activity, alcohol, violence, adolescents, Zambia

## Abstract

There is scanty information on correlates for psychosocial distress in Zambia. Secondary analysis was conducted using the data collected in 2004 in Zambia during the global school-based health survey to determine the prevalence and correlates for psychosocial distress. Logistic regression analyses were used to estimate magnitudes of associations between exposure factors and the outcome, while the Yates’ corrected Chi-squared test was used to compare proportions at the 5% significance level. A total of 2257 students participated in the survey of which 54.2% were males. Males were generally older than females (*p* < 0.001). Significantly, more females than males were bullied (*p* = 0.036), involved in a fight (*p* = 0.019), and consumed alcohol (*p* = 0.012). Psychosocial distress was detected in 15.7% of the participants (14.4% of males and 16.8% of females). Age <14 years, male gender, parental support for males, and having close friends were protective factors against psychosocial distress. Risk factors for psychosocial distress were being bullied, involvement in a fight, alcohol consumption, being physically active, and parental support. The prevalence of psychosocial distress among adolescents in Zambia appears to be common. There is a need to validate the psychosocial distress indicators that were used in the current study.

## Introduction

Psychosocial distress is a common health problem worldwide with varying rates (1) and it starts during childhood and adolescent (2). Prevalence rates for psychosocial distress varied between studies partly because different studies used different definitions for psychosocial distress. In some studies, psychosocial distress was assessed using four mental health indicators: loneliness, anxiety or worried, sadness, and suicide plan (3, 4). In another study, Hong et al. (5) defined psychosocial distress using five indicators: alcohol intoxication, drug use, suicidal behavior, depression, and loneliness. In yet another study, psychosocial distress was defined as a composite measure of depression, self-perceived stress, neuroticism, and dissatisfaction (6). Stevenson et al. (7) calculated the score for psychosocial distress by summing each respondent’s answers to the 29 SRQF questions. In a school-based survey in Iran, psychosocial distress was detected in 71.2% of boys and 63.3% of girls (8). Meanwhile, rates of 2.0% among boys and 3.0% among girls have been reported in Thailand (9).

Protective factors associated with psychosocial distress include living with both parents and good parent-teen connectedness. Risk factors for distress include having sex with two or more sex partners and substance use. In a study in Ethiopia in which 93% of the students were of age 15–19 years, Shiferaw et al. (10) found that living with both biological parents and good parent-teen connectedness were related to better psychosocial health. Page and Hall (4) reported that psychosocial distress was associated with having sex or having sex with two or more people among adolescents in sub-Saharan African countries including Botswana, Kenya, Namibia, Uganda, Zambia, and Zimbabwe. Using data obtained in Global School-Based Student Health Surveys in Philippines, China, Chile, and Namibia, Page et al. (11) found that adolescent substance use (lifetime use of alcohol, tobacco, and other drugs) was a risk factor for psychosocial distress.

There is scanty information on the correlates for psychosocial distress in Zambia, and the present study was aimed to determine the prevalence and correlates for psychosocial distress among in-school adolescents in order to contribute to the knowledge base for use to design interventions to curb this psychosocial problem among adolescents. Since the study was exploratory, no hypotheses could be tested and hoped that hypotheses could be generated from this study.

## Materials and Methods

Secondary analysis was conducted using the data (12) collected in 2004 in Zambia during the global school-based health survey (GSHS). A detailed description of the GSHS methodology that was used in Zambia is described elsewhere (13). The GSHS was developed by the World Health Organization (WHO) in collaboration with UNICEF, UNESCO, and UNAIDS with technical assistance from CDC, Atlanta, GA, USA. The main purpose of the GSHS is to obtain health and social behavioral data primarily among 13- to 15-year-old school-going adolescents. In Zambia, this age group was mostly in grades 7–10. Participants were randomly selected using a two-stage cluster sampling technique. In the first stage of sampling, 50 schools were randomly selected from 4621 government schools with probability proportional to their enrollment size. A total of 47 schools participated in the survey, giving a school response rate of 94%. At the second stage of sampling, classes were randomly selected and all students in selected classes irrespective of their ages were requested to participate in the survey. Out of 3021 eligible students, 2257 students participated in the survey giving a student response rate of 75%. The overall response rate was 70%.

### Ethical considerations

Ethical review and approval was granted by the Ministries of Health and Education. Permission to collect the data from the students was obtained from head teachers. Students were informed that they were free not to participate in the survey, and that they were free not to answer some questions or withdraw from the survey if they chose to participate the survey. The questionnaire did not have personal identifiers. Teachers were excused from classes during the administration of the questionnaire and did not have access to completed questionnaires.

### Data collection

Data were collected using a modified GSHS questionnaire. The 54 core questions in the GSHS questionnaire were on age, sex, weight, height, dietary behavior, household food security, personal hygiene, water, physical violence, injuries, bullying, personal safety, feelings, and friendship. The Zambia GSHS questionnaire also included 35 questions on alcohol abuse, drug abuse, sexual behavior and HIV/AIDS, physical activity, leisure time, and experiences at school (14). The questionnaire was filled-in by the students by recording their responses directly on a computer-scannable response sheet.

### Variables

The outcome variable “Psychosocial distress” was assessed using four mental health indicators: loneliness, anxiety or worried, sadness, and suicide plan based on previous research on a similar school-going adolescent population (9). A respondent with three or four of the four mental health indicators was declared as having psychosocial distress. The independent variables were age, sex, bullied, fight, alcohol, activity, parental support and supervision, and friendship. Table 1 shows the variables that were considered in the analysis.

**Table 1:** **Variables considered in the analysis**.

Variable	Description/question	Coded	Recoded
**Dependent**
Psychosocial distress	Assessed using four mental health measures	0–4	1 = 3 or 4
			0 ≤ 3
Loneliness	During the past 12 months, how often have you felt lonely?	1 = Never	1 = Most of the time or always
		2 = Rarely	2 = Never, rarely or sometimes
		3 = Sometimes	
		4 = Most of the time	
		5 = Always	
Worried	During the past 12 months, how often have you been so worried about something that you could not sleep at night?	1 = Never	1 = Most of the time or always
		2 = Rarely	2 = Never, rarely or sometimes
		3 = Sometimes	
		4 = Most of the time	
		5 = Always	
Sadness	During the past 12 months, did you ever feel so sad or hopeless almost every day for 2 weeks or more in a row that you stopped doing your usual activities?	1 = Yes	1 = Yes
		2 = No	2 = No
Suicide plan	During the past 12 months, did you make a plan about how you would attempt suicide?	1 = Yes	1 = Yes
		2 = No	2 = No
**Independent**
Age	How old are you?	1 = 11 years old or younger	1 = 14 years or younger
		2 = 12 years old	2 = 14 years
		3 = 13 years old	3 = 15 years
		4 = 14 years old	4 = 16 years or older
		5 = 15 years old	
		6 = 16 years or older	
Sex	What is your sex?	1 = Male	1 = Male
		2 = Female	2 = Female
Bullied	During the past 30 days, on how many days were you bullied?	1 = 0 days	1 = 1–30 days
		2 = 1–2 days	2 = 0 days
		3 = 3–5 days	
		4 = 6–9 days	
		5 = 10–19 days	
		6 = 20–29 days	
		7 = All 30 days	
Fight	During the past 12 months, how many times were you in a physical fight?	1 = 0 times	1 = 1 or more times
		2 = 1 time	2 = 0 times
		3 = 2 or 3 times	
		4 = 4 or 5 times	
		5 = 6 or 7 times	
		6 = 8 or 9 times	
		7 = 10 or 11 times	
		8 = 12 or more times	
Alcohol	During the past 30 days, on how many days did you have at least one drink containing alcohol?	1 = 0 days	1 = 1 or more days
		2 = 1 or 2 days	2 = 0 days
		3 = 3–5 days	
		4 = 6–9 days	
		5 = 10–19 days	
		6 = 20–29 days	
		7 = All 30 days	
Activity	During the past 7 days, on how many days were you physically active for a total of at least 60 minutes per day?	1 = 0 days	1 = 4–7 days
		2 = 1 day	2 < 4 days
		3 = 2 days	
		4 = 3 days	
		5 = 4 days	
		6 = 5 days	
		7 = 6 days	
		8 = 7 days	
Parental supervision	During the past 30 days, how often did your parents or guardians really know what you were doing with your free time?	1 = Never	1 = Most of the time or always
		2 = Rarely	2 = Never, rarely or sometimes
		3 = Sometimes	
		4 = Most of the time	
		5 = Always	
Parental support	During the past 30 days, how often did your parents or guardians understand your problems and worries?	1 = Never	1 = Most of the time or always
		2 = Rarely	2 = Never, rarely or sometimes
		3 = Sometimes	
		4 = Most of the time	
		5 = Always	
Friendship	How many close friends do you have?	1 = 0	1 = 1 or more
		2 = 1	2 = 0
		3 = 2	
		4 = 3 or more	

### Data analysis

A Statistical Package for Social Sciences (SPSS) version 17.0 was used in the analysis. Weighted analyses were conducted using the following weights in order to account for differences in response rates:

W=W1∗W1∗f1∗f2∗f3∗f4

where W1 = the inverse of the probability of selecting the school, W2 = the inverse of the probability of selecting the classroom within the school, fl = a school-level non-response adjustment factor calculated by school size category (small, medium, large), f2 = a class-level non-response adjustment factor calculated for each school, f3 = a student-level non-response adjustment factor calculated by class, and f4 = a post stratification adjustment factor calculated by grade.

Proportions were compared using the Yates’ corrected Chi-squared test in 2 × 2 tables and the Pearson’s Chi-squared test was used in higher contingency tables. The level of statistical significance was set at the 5% level. Logistic regression analyses were conducted using a backward variable selection method to determine associations between selected independent variables and psychosocial distress. Unadjusted odds ratios (OR) and adjusted odds ratios (AOR) together with their 95% confidence intervals (CI) were used to estimate magnitudes of associations.

## Results

A total of 2257 students participated in the survey of which 54.2% were males. About one-third (30.4%) of the participants were of age 16 years or older. Males were generally older than females (*p* < 0.001) with 61.3% of males and 48.3% of females being of age 15 years or older. Psychosocial distress was detected in 15.7% of the participants (14.4% of males and 16.8% of females). These results are shown in Table 2.

**Table 2:** **Sample description**.

Factor	Total *n* (%)	Male *n* (%)	Female *n* (%)	*p* Value
**Age**
<14	472 (26.6)	159 (20.6)	269 (30.0)	<0.001
14	418 (19.5)	174 (18.1)	233 (21.7)	
15	552 (23.5)	279 (25.3)	258 (22.2)	
16+	747 (30.4)	410 (36.0)	328 (26.1)	
**Sex**
Male	1052 (54.2)	–	–	
Female	1101 (45.8)			
**Bullied**
Yes	999 (63.1)	451 (60.4)	495 (65.2)	0.036
No	642 (36.9)	330 (39.6)	290 (34.8)	
**Fight**
Yes	1086 (51.7)	476 (49.3)	553 (54.0)	0.019
No	1134 (48.3)	561 (50.7)	529 (46.0)	
**Alcohol**
Yes	655 (46.8)	267 (43.1)	358 (49.9)	0.012
No	835 (53.2)	400 (56.9)	407 (50.1)	
**Activity**
Yes	358 (18.0)	150 (16.0)	189 (20.0)	0.093
No	1639 (82.0)	776 (84.0)	793 (80.0)	
**Parental supervision**			
Yes	619 (31.7)	282 (31.3)	310 (32.8)	0.996
No	1238 (68.3)	562 (68.7)	615 (67.2)	
**Parental support**			
Yes	676 (35.2)	297 (33.6)	352 (37.4)	0.157
No	1171 (64.8)	551 (66.4)	565 (62.6)	
**Friendship**			
Yes	1848 (84.3)	865 (85.1)	912 (84.4)	0.966
No	342 (15.7)	154 (14.9)	162 (15.6)	
**Psychosocial distress score**			
0	391 (20.5)	208 (23.1)	178 (18.4)	0.153
1	678 (35.1)	318 (34.5)	334 (35.6)	
2	521 (28.7)	231 (28.0)	268 (29.2)	
3	225 (12.3)	96 (11.3)	115 (13.1)	
4	61 (3.3)	26 (3.1)	33 (3.7)	

*n, unweighted frequencies; (%), weighted percent*.

Tables 3 and 4 show factors that were associated with psychosocial distress. In bivariate analyses, all the factors that were considered in the analysis were significantly associated with psychosocial distress. Male participants were 2% (AOR = 0.8, 95% CI [0.96, 1.00]; *p* = 0.017) less likely to have psychosocial distress compared to females. Overall, participants of age <14 years were 37% (AOR = 0.63; 95% CI [0.60, 0.65]) less likely to have psychosocial distress compared to participants of age 16 years or older. While male participants of age 14 years were 20% (AOR + 0.80; 95% CI [0.75, 0.86]) less likely to have psychosocial distress, female participants were 46% (AOR = 1.46; 95% CI [1.39, 1.52]) more likely to have psychosocial distress compared to participants of age 16 years or older.

**Table 3:** **Factors associated with psychosocial distress in bivariate analysis**.

Factor	Total OR (95% CI)	Male OR (95% CI)	Female OR (95% CI)
**Age**
<14	0.79 (0.78, 0.81)	0.81 (0.79, 0.84)	0.73 (0.72, 0.75)
14	1.06 (1.04, 1.07)	0.88 (0.85, 0.91)	1.23 (1.20, 1.26)
15	1.02 (1.00, 1.04)^a^	1.15 (1.13, 1.18)	0.89 (0.87, 0.92)
16+	1	1	1
**Sex**
Male	0.91 (0.90, 0.92)	–	–
Female	1		
**Bullied**
Yes	1.65 (1.63, 1.67)	1.49 (1.47, 1.52)	1.85 (1.81, 1.89)
No	1	1	1
**Fight**
Yes	1.46 (1.45, 1.48)	1.43 (1.41, 1.45)	1.48 (1.45, 1.50)
No	1	1	1
**Alcohol**
Yes	2.17 (2.14, 2.20)	1.98 (1.95, 2.02)	2.45 (2.40, 2.50)
No	1	1	1
**Activity**
Yes	1.36 (1.35, 1.38)	1.29 (1.26, 1.31)	1.91 (1.84, 1.97)
No	1	1	1
**Parental supervision**			
Yes	1.03 (1.02, 1.04)	0.92 (0.90, 0.93)	1.13 (1.11, 1.15)
No	1	1	1
**Parental support**			
Yes	1.05 (1.04, 1.06)	0.95 (0.94, 0.97)	1.13 (1.11, 1.15)
No	1	1	1
**Friendship**
Yes	0.95 (0.93, 0.96)	0.94 (0.92, 0.95)	0.94 (0.92, 0.96)
No	1	1	1

*^a^p = 0.048*.

*OR, odds ratio; (95% CI), 95% confidence interval*.

**Table 4:** **Factors associated with psychosocial distress in multivariate analysis**.

Factor	Total AOR (95% CI)	Male AOR (95% CI)	Female AOR (95% CI)
**Age**
<14	0.63 (0.60, 0.65)	0.71 (0.68, 0.75)	0.59 (0.56, 0.62)
14	1.15 (1.11, 1.19)	0.80 (0.75, 0.86)	1.46 (1.39, 1.52)
15	1.03 (1.00, 1.06)^a^	1.02 (0.98, 1.07)	1.01 (0.97, 1.06)
16+	1	1	1
**Sex**
Male	0.98 (0.96, 1.00)^b^	–	–
Female	1		
**Bullied**
Yes	1.18 (1.16, 1.21)	1.26 (1.22, 1.30)	1.15 (1.10, 1.19)
No	1	1	1
**Fight**
Yes	1.25 (1.23, 1.28)	1.14 (1.11, 1.17)	1.35 (1.31, 1.39)
No	1	1	1
**Alcohol**
Yes	2.22 (2.17, 2.27)	2.31 (2.24, 2.39)	2.21 (2.13, 2.29)
No	1	1	1
**Activity**
Yes	1.25 (1.22, 1.28)	1.18 (1.14, 1.22)	1.35 (1.31, 1.39)
No	1	1	1
**Parental supervision**			
Yes	0.84 (0.83, 0.86)	0.69 (0.67, 0.71)	–
No	1	1	
**Parental support**			
Yes	1.20 (1.18, 1.22)	1.04 (1.01, 1.07)	1.47 (1.43, 1.51)
No	1	1	1
**Friendship**
Yes	0.90 (0.88, 0.93)	0.91 (0.88, 0.95)	0.87 (0.83, 0.90)
No	1	1	1

*^a^p = 0.089*.

*^b^p = 0.017*.

*AOR, adjusted odds ratio; (95% CI), 95% confidence interval*.

Being bullied was significantly associated with psychosocial distress (AOR = 1.26; 95% CI [1.22, 1.30]) for males and AOR = 1.15; 95% CI [1.10, 1.19]) for females). Male (AOR = 1.14; 95% CI [1.11, 1.17]) and female (AOR = 1.35; 95% CI [1.31, 1.39]) participants who were involved in a fight were more likely to have psychosocial distress compared to participants who were not involved in a fight. Alcohol consumption was significantly associated with psychosocial distress. Participants who consumed alcohol were about twice (AOR = 2.31; 95% CI [2.24, 2.39] for males and AOR = 2.21; 95% CI [2.13, 2.29] for females) more likely to have psychosocial distress compared to participants who did not consume alcohol.

Participants who were physically active for a period of 4–7 days in a week was more likely to have psychosocial distress (AOR = 1.18; 95% CI [1.14, 1.22] for males and AOR = 1.35; 95% CI [1.31, 1.39] for females).

Concerning social support, male participants with parents or guardians who most of the time or always understood their problems and worries were 4% (AOR = 1.04; 95% CI [1.01, 1.07]) more likely to have psychosocial distress compared with participants with parents who never, rarely, or sometimes understood their problems or worries. Meanwhile, female participants with parents or guardians who most of the time or always understood their problems or worries were 47% (AOR = 1.47, 95% CI [1.43, 1.51]) more likely to have psychosocial distress compared with participants with parents or guardians who never, rarely, or sometimes understood their problems or worries.

Male participants with parents or guardians who most of the time or always really knew what they were doing with their free time were 31% (AOR = 0.69; 95% CI [0.67, 0.71]) less likely to have psychosocial distress compared with participants with parents who never, rarely, or sometimes knew what they were doing during their free time.

Having close friends was negatively associated with psychosocial distress. Overall, participants who had close friends were 10% (AOR = 0.90; 95% CI [0.88, 0.93]) less likely to have psychosocial distress compared with participants who did not have close friends.

## Discussion

The current paper is the first one to document the prevalence and correlates for psychosocial distress among adolescents in Zambia. Analyses were stratified by sex because males and females respond to stimuli for psychosocial distress differently. In this study, psychosocial distress was detected in 15.7% of the participants (14.4% of males and 16.8% of females). Factors that were significantly associated with psychosocial distress were age, being bullied, fight, alcohol, activity, parental support, parental supervision, and friendship.

The prevalence of 15.7% in the present study was lower than the 67.7% (71.2% of males and 63.3% of girls) observed in Iran (8). However, the rate in the current study was higher than the one reported in Thailand (9) of 2.5% overall (2.0% among boys and 3.0% among girls). The differences in the rates of psychosocial distress are partly due to differences in the definitions for psychosocial distress that were used in the studies. In the Iran study, seven questions regarding worthlessness, angriness, anxiety, insomnia, confusion, sadness, and worried problems were used to screen students for psychosocial distress. Respondents with at least four of the seven items (57% or more) in the Iranian were considered distressed. Meanwhile, in the present study and in Thailand, four mental health indicators: loneliness, anxiety or worried, sadness, and suicide plan were used to define psychosocial distress and respondents with three or four out of four items (75% or more) were considered distressed. This difference in cut off points for distress may partly explain the observed different rates of psychosocial distress.

In the current study, while both males and females aged <14 years were less likely to have psychosocial distress, at the age of 14 years males continued to be less likely to have distress while females became more likely to be distressed compared to persons aged 16 years or older. Kaltiala-Heino et al. (15) observed that early pubertal timing is associated with increased mental health problems. Since females mature faster than males (16), in the early adolescence stage, females will be more likely to be psychosocially distressed compared to males in the same age group. In a related study, Ge et al. (17) observed that gender differences in depressive symptoms occurred during eighth grade and noted the interaction between the pubertal transition and psychosocial factors in depressive experiences.

The association of sex with psychosocial distress has not been consistent. The difference in rates of psychosocial distress between males and females may be due to differences in copying strategies to stressors. Lawrence et al. (18) observed that males tend to detach themselves from emotions of situations and be emotionally inhibited than females. Although in Iran, boys had a higher rate of distress than girls (8), the finding in the current study suggests that females had a higher risk for distress than males. Results from another study conducted in Iran (19) accord the findings from the current study that girls are more likely to have distress than boys. Several factors may contribute to the inconsistency of the association between sex and psychosocial distress and include temporal and geographical characteristics, differences in methods and tools for screening and diagnosis, difference in classification system, age groups of participants, and small sample size (19, 20). Overall, older participants were more likely to have psychosocial distress in the current study and this finding is similar to that obtained in Iran that reported a significantly higher mean age for students with psychosocial distress than those without (8). It is possible that younger adolescents may still be regarded as children and the community may not be as strict to them as to the older adolescents who are expected to behave like adults. Hence, older adolescents may have difficulties coping with stimuli, such as changing cultural values and societal roles. Older adolescents may also encounter dating disappointments, sexual, physical, and emotional abuses. Interventions to control distress should be targeted to younger age groups (<14 years) before they have psychosocial distress.

Violence has been associated with psychosocial distress in the current study and similar findings are reported elsewhere. Among obese youth in Flanders, Belgium, bullying was associated with lower psychosocial health among obese youth (21). In Thailand, Pengpid and Peltzer (9) also reported a significant relationship between having been bullied and psychosocial distress. Participants who were involved in a fight in the present study were more likely to have psychosocial distress compared to participants who were not involved in a fight. It is possible that involvement in a fight might have induced the distress in the participants. It is also likely that participants got involved in a fight because of their temperamental state resulting from the distress. Because of the cross-sectional design of the study, we are unable to determine the pathway of the association.

Life styles-related factors have been associated with psychosocial distress in the current study. Alcohol consumption was significantly associated with psychosocial distress. Participants who consumed alcohol were about twice more likely to have psychosocial distress compared to participants who did not consume alcohol. It is not possible to determine the effect and cause in a cross-sectional study. It is possible that distressed persons may turn to drinking alcohol with the hope of “drinking away the problem” and it is also possible that alcohol consumption can lead to psychosocial distress. Furthermore, a third variable can explain the observed relationship between alcohol and psychosocial distress. Participants in the current study who were physically active were more likely to have psychosocial distress. It is unlikely that being physically active may lead to psychosocial distress. To the contrary, turning to sport may be a way to deal with the distress. Further studies considering more powerful study designs, such as case control or cohort studies, should be conducted to test the associations between life-style factors (such as alcohol consumption and physical activity) and psychosocial distress.

Concerning social support, participants with parents or guardians who most of the time or always understood their problems and worries were more likely to have psychosocial distress compared with participants with parents who never, rarely, or sometimes understood their problems or worries. It is unlikely that parental support could cause psychosocial distress in their children. To the contrary, psychosocial distress might have been the problem that could have made parents to socially support their children to deal with their problems. Male participants with parents or guardians who most of the time or always knew what they were doing with their free time were less likely to have psychosocial distress compared to male participants who had parents or guardians who did not know what they were doing with their free time. Parents should support their children and discuss their problems to avoid distress. Having close friends was protective against psychosocial distress. Close friends should always be there for their friends to share their worries and it would in turn avoid the distress.

The main limitation of this study is its cross-sectional design, which does not demonstrate the causality of association between the assumed exposure factors and psychosocial distress. We did not find records of validation of the psychosocial distress indicators that were used in the current study, apart from the fact that the indicators have been used in previous studies. Hence, we do not know the magnitude of the bias and its direction that could have been introduced in our study. The strength of present study lies in its large sample size and representativeness of the in-school-going students. The findings may not be generalized to the out-school adolescents as the occurrence of psychosocial distress may differ between in- and out-school adolescents. However, there might be minimum bias because of high-enrollment rates in Zambia. However, an overall non-response rate of 30% may cause concern with regard to the validity of our results. Our results will not be valid to the extent the non-respondents differed from the students who participated in the survey. Information on the characteristics of non-respondents was not available to enable assessment of the magnitude and direction of the bias that would have been introduced. To our knowledge, this is the first study in Zambia to examine associations for psychosocial distresses.

In conclusion, the prevalence of psychosocial distress among adolescents in Zambia appears to be common. There is a need to validate the psychosocial distress indicators that were used in the current study. If there will be need to consider more indicators to refine the definition for psychosocial distress, prevalence studies will need to be conducted to establish the magnitude of psychosocial distress and its correlates in order to generate hypotheses on psychosocial distress in our population. These hypotheses would then be tested using more powered study designs, such as cohort studies or randomized field trials, in order to accrue more evidence toward causality.

## Conflict of Interest Statement

The authors declare that the research was conducted in the absence of any commercial or financial relationships that could be construed as a potential conflict of interest.
